# The impact of EV71 vaccination program on hand, foot and mouth disease in Zhejiang Province, China: A negative control study

**DOI:** 10.1016/j.idm.2023.09.001

**Published:** 2023-09-05

**Authors:** Dashan Zheng, Lingzhi Shen, Wanqi Wen, Feng Ling, Ziping Miao, Jimin Sun, Hualiang Lin

**Affiliations:** a Department of Epidemiology, School of Public Health, Sun Yat-sen University, Guangzhou, Guangdong, 510080, China; b Zhejiang Provincial Center for Disease Control and Prevention, Hangzhou, Zhejiang, 310051, China

**Keywords:** Hand, foot, and mouth disease (HFMD), Bayesian structure time series model, Enterovirus A71 (EV71) vaccine, Negative control outcome

## Abstract

**Objective:**

To estimate the potential causal impact of Enterovirus A71 (EV71) vaccination program on the reduction of EV71-infected hand, foot, and mouth disease (HFMD) in Zhejiang Province.

**Methods:**

We utilized the longitudinal surveillance dataset of HFMD and EV71 vaccination in Zhejiang Province during 2010–2019. We estimated vaccine efficacy using a Bayesian structured time series (BSTS) model, and employed a negative control outcome (NCO) model to detect unmeasured confounding and reveal potential causal association.

**Results:**

We estimated that 20,132 EV71 cases (95% CI: 16,733, 23,532) were prevented by vaccination program during 2017–2019, corresponding to a reduction of 29% (95% CI: 24%, 34%). The effectiveness of vaccination increased annually, with reductions of 11% (95% CI: 6%, 16%) in 2017 and 66% (95% CI: 61%, 71%) in 2019. Children under 5 years old obtained greater benefits compared to those over 5 years. Cities with higher vaccination coverage experienced a sharper EV71 reduction compared to those with lower coverage. The NCO model detected no confounding factors in the association between vaccination and EV71 cases reduction.

**Conclusions:**

This study suggested a potential causal effect of the EV71 vaccination, highlighting the importance of achieving higher vaccine coverage to control the HFMD.

## 1 Introduction

Hand, foot, and mouth disease (HFMD) is a common infectious disease primarily affecting children aged below 5 years (Yang et al., 2017). Over the last few decades, the Asian-Pacific region, including Vietnam, Singapore, and China, had experienced the most significant epidemic of HFMD (Gonzalez et al., 2019; Huang et al., 2018). HFMD could be caused by various enteroviruses, including Coxsackievirus A16 (CA16) and Enterovirus A71 (EV71), with EV71 being recognized as the primary pathogen associated with severe illness or fatality (Chang et al., 2018; Ji et al., 2019). Since its reclassification as a notifiable infectious disease in May 2008, HFMD has become the most commonly reported infectious disease in China (Xing et al., 2014). Thus, to prevent HFMD, several inactivated EV71 vaccines were developed in China and licensed for administration (Li et al., 2014; Zhu et al., 2014). Although previous phase Ⅰ-Ⅲ clinical trials have demonstrated certain efficacy of the vaccine, there were still numerous reports of inconsistency in vaccine effectiveness between the preclinical and post-market stages (Lopalco et al., 2015). Therefore, conducting post-market surveillance (phase IV clinical trials) for the EV71 vaccine is imperative.

In recent years, several hospital-based studies have evaluated the effectiveness of the EV71 vaccine in phase IV clinical trials (Wang et al., 2021). For example, one case control study in Henan estimated that the vaccine effectiveness was 85.4% for fully vaccinated participants and 63.1% for partly vaccinated (Li et al., 2019). Moreover, a few studies also examined the real-world effectiveness of the EV71 vaccination program, providing a more accurate and comprehensive assessment of the vaccination's impact on the general population (Du et al., 2021). For instance, one study in Chengdu also estimated that the EV71 vaccination program could reduce 60% EV71-infected HFMD cases (Head et al., 2020).

However, the existing real-world studies encountered challenges in detecting confounding bias (Jackson et al., 2013; Lipsitch et al., 2016). For example, previous studies estimated the effectiveness of the EV71 vaccine program by comparing the trends in HFMD before and after the implementation of the vaccination program, without considering unknown or unmeasured confounding factors, such as changes in sanitation and economic levels during the study period (Head et al., 2020; Xiao et al., 2022). If these confounding factors changed during the study period, their impact on HFMD cannot be determined solely by examining the time series data (Flanders et al., 2017). Therefore, there is still a lack of causal evidence on the effect of EV71 vaccination among real-world population.

To address the research gap, this study collected high-quality surveillance data from Zhejiang Province, and employed a Bayesian structure time series (BSTS) model to accurately assess the effectiveness of the EV71 vaccination program. Furthermore, we developed a negative control outcome (NCO) model to detect the unmeasured confounding and reveal the potential causal association between vaccination program and the number of EV71 cases.

## 2 Methods

### 2.1 Study area and population

**Fig. 1:**
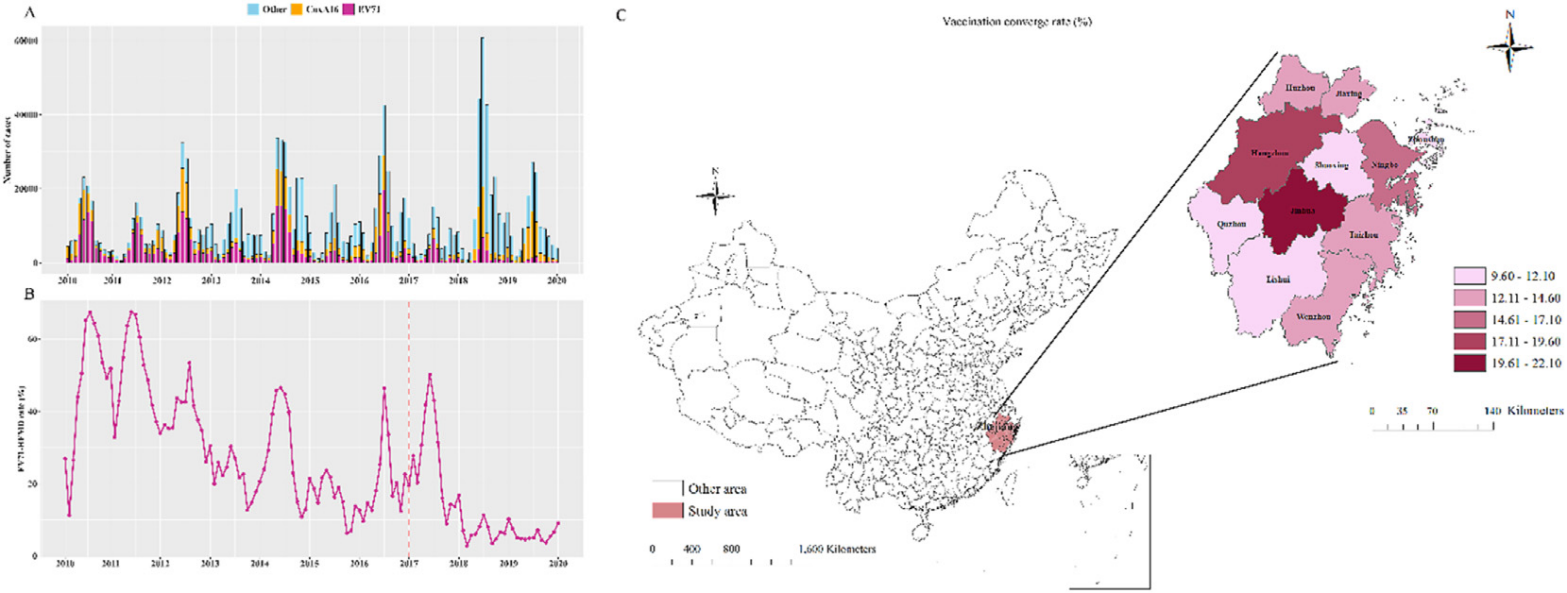
The trends of EV71, Cox A16 and other enterovirus cases (A); The proportion of EV71-infected cases among all HFMD cases (B). The study area and the geographical distribution of EV71 vaccine coverage rate in 11 cities of Zhejiang Province, China, 2017–2019 (C).

Zhejiang Province is located in the southeast coast of China (118°E−123°E, 27°N-32°N), with humid air, mild climate and developed economy (Fig. 1C). Covering an area of 101,800 square kilometers and 11 cities, Zhejiang is one of the most densely populated provinces in China. In addition, the incidence of HFMD in Zhejiang Province has ranked the top three (Wu et al., 2022), with a rapid increase from 2009 to 2016, making it the highest among notifiable diseases and predominantly associated with the EV71 serotype (Lu et al., 2019).

### 2.2 Data collection

The HFMD cases confirmed by China's national diagnostic standards in hospital must be reported by medical personnel through the National Notifiable Infectious Diseases Reporting Information System (NNIDRIS). The surveillance data of HFMD cases in Zhejiang Province 2010–2019 was collected from NNIDRIS, including gender, age, education level, location and the date of incidence.

The etiological information was collected from Zhejiang Province Center for Disease Control and Prevention (CDC). Specifically, a fraction of recorded HFMD sample that were selected randomly from monthly reported HFMD cases, were sent to the laboratory for nucleic acid detection of CA16, EV71 and other enterovirus, using polymerase chain reaction or virus isolation (Wu et al., 2022). Real-time reverse transcription polymerase chain reaction (RT-PCR) was used to detect all samples. Considering that not all the recorded HFMD cases were distinguished through etiological detection, we employed the following method to estimate the monthly counts of HFMD cases associated with EV71:

EV71 cases = HFMD cases × EV71 positive rate from etiological detected

In order to estimate the effect of EV71 vaccine among different age population, we also divided the HFMD cases into <3 years old, 3–5 years old and >5 years old. Also, we separately estimated the effect of EV71 vaccine in both nursery and scattered children. For those different groups, we used the above methods to estimate EV71 cases, that is, we first calculated the proportion of EV71 in the examined samples of the group in each month (Table S1), and then estimated the monthly number of EV71 cases of the group according to this proportion and the total number of cases in the group in that month.

The vaccination information was also collected from Zhejiang CDC. The vaccination schedule consists of two doses given 28 days apart, which was initiated in August 2016. We estimated the vaccination coverage rate for each of the 11 cities and divided them into high, moderate and low vaccination coverage cities based on the tertiles of these 11 vaccination coverage rates. The vaccination coverage rate was calculated by following equation:

Vaccination coverage rate = The number of vaccinated people / The number of people under 16 years old

### 2.3 Statistical analysis

To investigate the effectiveness of the EV71 vaccination program, we utilized a two-stage analytical approach.

We initially employed a counterfactual framework and utilized a BSTS model to forecast the occurrence of EV71-infected HFMD. Subsequently, the difference between predicted and observed cases was used to estimate the vaccination effect.

The BSTS model is a state-space model for time-series data combined with Bayesian approach, enabling the estimation of how the impact that can be attributed to a specific factor change over time (Vizzotti et al., 2016). It allows for the incorporation of empirical priors on the parameters within a fully Bayesian framework and has been specifically utilized for estimating the impact of interventions in the field of epidemiology (Liu et al., 2021). In this study, due to the HFMD vaccination starting from the end of August 2016 with only 28,648 vaccinations by the end of December 2016 in Zhejiang Province, we set the 2010–2016 as the training set to predict the number of EV71 cases that would occurred in the absence of vaccination during 2017–2019. All the monthly cases were log-converted due to the great various of HFMD between different months.

To simulate the effect of EV71 vaccination program, the BSTS model can be defined in terms of a pair of equations:

(1) $$\mathrm{Log}\left(Y_{t}\right)=Z_{t}\alpha_{t}+\varepsilon_{t},\varepsilon_{t}∼N\left(0,H_{t}\right)$$

(2) $$\alpha_{t+1}=T_{t}\alpha_{t}+R_{t}\eta_{t},\eta_{t}∼N\left(0,Q_{t}\right)$$

Where a latent d-dimensional state vector $\alpha_{t}$ can be explicitly written as following:

(3) $$\alpha_{t}={\left({Year}_{t},{Month}_{t},{Month}_{t-1},\ldots ,{Month}_{t-10},\beta_{1t},\beta_{2t},...\right)}^{T}$$

Equation (1) was the observation equation, which established a connection between the observed data $Y_{t}$ (the number of EV71 cases at time t) and the unobserved latent state $\alpha_{t}$. Additionally, $Z_{t}$ was a d-dimensional vector of coefficients applied to the state and $\varepsilon_{t}$ was the normally distributed error term of the observations. Equation (2) was the transition equation, which described the evolution of the latent state $\alpha_{t}$ over time. Specifically, $T_{t}$ was the transition matrix, $R_{t}$ represented the control matrix and $\eta_{t}$ meant system error. The matrices $Z_{t}$, $T_{t}$ and $R_{t}$ typically contained a mix of known values (frequently 0 and 1) and unknown parameters (Brodersen et al., 2015; Scott et al., 2014).

Equation (3) denoted that the state vector $\alpha_{t}$ considered in our study is composed by trend, seasonal, and regression effects components. To be specific, ${Year}_{t}$ used to control long-term trend, ${Month}_{t}$ was used to control seasonality (12 per year) and $\beta_{it}\left(i=1,2,...\right)$ were the regression coefficients that allows a set of external factors to contribute to the prediction.

We used Kalman filters for time series forecasting and considered spike and slab prior for the selection of optimal covariates. The final prediction was simulated from the posterior distribution using the Markov Chain Monte Carlo (MCMC) algorithm, and Bayesian model averaging was employed to smooth over a large number of potential models. The number of prevented cases from vaccine administration was represented by subtracting the predicted number of cases based on counterfactual analysis from the actual number of cases monitored by the disease control center. The relative reduction was calculated by dividing the number of prevented cases by the expected number of cases (Vizzotti et al., 2016).

Secondly, considering the potential confounding bias due to some unknown or unmeasured factors, such as economic factors and healthcare policies, we conducted a NCO analysis to detect residual confounding bias (Fig. 4). The NCO is a variable known not to be causally affected by the intervention of interest (Sofer et al., 2016). Specifically, the presence of an association between the NCO and treatment can be examined to determine whether there is residual confounding bias in the study. Fig. 4 showed the association between EV71 vaccination, the prevention of EV71-infected HFMD, the negative control outcome and the unmeasured confounding. The inferred equations were as follows:

(4) $$\begin{array}{c} E\left[Y|A,U\right]=\beta_{Y0}+\beta_{YA}A+\beta_{YU}U \end{array}$$

(5) $$\begin{array}{c} E\left[X|U\right]=\beta_{X0}+\beta_{XU}U \end{array}$$

(6) $$\begin{array}{c} E\left[U|A\right]=\beta_{U0}+\beta_{UA}A \end{array}$$

Equation (4) represents the relationship between observation outcome Y (The EV71-infected cases), exposure A (The vaccination program) and residual confounding U (economic factors and healthcare policies), equation (5) represents the relationship between residual confounding U and negative control outcome X, equation (6) represents the relationship between residual confounding U and exposure A. If U was known to be confounding, the coefficient $\beta_{YA}$ of A in (4) was the true causal association of A-Y.

In equations (4), (5) of the observation results and the negative control results, E[U|A] substituted for U of equation (6) can be obtained:

(7) $$\begin{array}{c} E\left[Y|A\right]=\beta_{Y0}+\beta_{YA}A+\beta_{YU}E\left[U|A\right]=\beta_{Y0}+\beta_{YA}A+\beta_{YU}\left(\beta_{U0}+\beta_{UA}A\right) \end{array}$$

(8) $$\begin{array}{c} E\left[X|A\right]=\beta_{X0}+\beta_{XU}E\left[U|A\right]=\beta_{X0}+\beta_{XU}\left(\beta_{U0}+\beta_{UA}A\right) \end{array}$$

The $\delta_{A}^{Y}{≜\beta }_{YA}+\beta_{YU}\beta_{UA}$ was the coefficient of A, where $\beta_{YU}\beta_{UA}$ was residual confounding bias associated with A-Y, and since U was unknown, we could not isolate the true causal effect from it. Additionally, since there was no causal association between A and X, the coefficient $\delta_{A}^{X}{≜\beta }_{XU}\beta_{UA}$ of A in equation (8) reflects the residual confounding bias of the association A-X (Flanders et al., 2017).

In fact, if association U–Y and association U-X are equivalent, then $\beta_{XU}=\beta_{YU}$*.* Therefore, $\delta_{A}^{X}$ was equivalent to association A-Y residual confounding bias ($\beta_{YU}\beta_{UA}$), and the true causal association $\beta_{YA}=\delta_{A}^{Y}-\delta_{A}^{X}$ exposed to observation outcome Y can be inferred using negative control outcome X (Shi et al., 2020; Sofer et al., 2016). If the association A-X was meaningless which means $\delta_{A}^{X}=0$, then the observed association A-Y was A true causal association (${\beta_{YA}=\delta }_{A}^{Y}$) (Sofer et al., 2016).

However, in reality, $\beta_{XU}$ cannot be completely equivalent to $\beta_{YU}$ (Arnold et al., 2016), thus, in order to ensure that $\beta_{XU}$ was approximately equal to $\beta_{YU}$, several criteria are used to select NCO: 1) NCO should be similar to EV71-infected HFMD in demographic characteristics such as age and sex, as well as in health status and exposure to risk factors for hand, foot and mouth disease. 2) NCO and EV71-infected HFMD should be exposed in similar environments, both of which should be affected by economic and medical levels. 3) The sample size of the NCO should be large enough to ensure statistical power. 4) NCO should not directly associate with EV71-infected cases (Richardson et al., 2015).

Thus, the scarlet fever was selected as the NCO (X) to detect the bias of unmeasured confounding factors (U) between EV71 vaccination (A) and the number of observed EV71 cases (Y). We included the monthly scarlet fever as NCO into the BSTS model and compared the predicted and observed cases from 2017 to 2019. When HFMD and scarlet fever are comparable ($\beta_{XU}\approx \beta_{YU}$.), and if there was no statistically significant reduction in scarlet fever, it indicated that $\beta_{YU}\beta_{UA}\approx 0$ (Lipsitch et al., 2010; Shi et al., 2020), then the unmeasured confounding (economic factors and healthcare policies) directly associated with scarlet fever was not exist, which effectively weakens the influence of unmeasured confounding and indicates that there was potential causal effect of EV71 vaccination program on EV71-infected HFMD. This derived equation was provided in the supplementary material.

#### 2.3.1 Sensitivity analysis

Several sensitivity analyses were performed to verify the stability of the results. Firstly, given that EV71 was the main cause of severe illnesses and deaths, we included all severe but untested individuals as EV71 cases, estimated monthly EV71 patients, and performed further analysis. Secondly, we excluded patients older than 12 years, 10 years and 8 years, respectively.

All the statistical analyses were conducted in R software (version: 4.1.1) and based on R packages “bsts” and “CausalImpact”. *P* values (two-tailed) less than 0.05 were considered statistically significant.

## 3 Results

### 3.1 Descriptive results

This analysis included 1,412,224 cases of HFMD in Zhejiang during the period of 2010–2019, which exhibited seasonal and biannual pattern. Among the reported cases, we estimated 379,501 were caused by EV71, 322,452 were attributed to CA16, and 710,271 were associated with other enteroviruses. Notably, we observed that the proportion of EV71 cases declined sharply after the vaccine implementation (Fig. 1B). For instance, the proportion of EV71 cases rate peaked at over 60% in the summer of 2010, whereas in 2019, it was almost below 10%.

Additionally, the vaccination doses of EV71 vaccines administered in 2016, 2017, 2018 and 2019 were 28,648, 369,409, 622,755 and 571,630, respectively. Based on the population under 16 years of age, we calculated the 2019 vaccination rates for each city, with the highest rate in Jinhua (22%) and lowest rate in Lishui (9.6%).

### 3.2 The fitting goodness of BSTS model

To assess the fitting goodness of the BSTS model, we utilized the 2010–2013 data as training set to simulate the EV71 cases during 2014–2016 (Fig. S1). We computed the RMSE, MAPE, and *R*^2^ metrics to evaluate the predictive accuracy of the model. Our results revealed that the BSTS model produced highly accurate predictions, with a MAPE of 4.6%, a RMSE of 0.43, and a *R*^2^ of 0.804, indicating a robust fit to the data.

### 3.3 The effectiveness of EV71 vaccination program in reducing EV71 cases

**Fig. 2:**
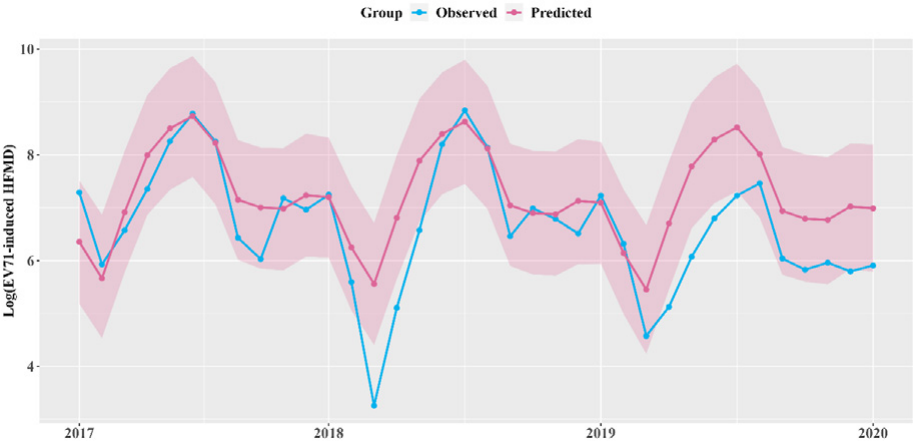
The comparison between monthly predicted counterfactual EV71-infected cases (pink solid line) and observed cases (blue solid line) from 2017 to 2019 in Zhejiang Province.

Since the implementation of the vaccine, we observed a decline in the number of detected cases compared to the predicted number (Fig. 2 & Table 1). Specifically, we estimated that 20,132 (95% CI: 16,733 to 23,532) cases of EV71 were prevented in Zhejiang Province between 2017 and 2019, corresponding to a reduction of 29% (95% CI: 24%–34%).

**Table 1:** The observed, predicted, prevented cases and the relative reduction of EV71-infected cases in Zhejiang Province, 2017–2019.

Year	Observed cases	Predicted cases	Prevented cases	Relative reduction
2017	23149	25925 (24680, 27170)	2776 (1531, 4021)	11% (6%, 16%)
2018	19819	23322 (22195, 24448)	3503 (2376, 4629)	15% (10%, 20%)
2019	7116	20970 (19912, 22027)	13854 (12796, 14911)	66% (61%, 71%)
2017–2019	50084	70216 (66817, 73616)	20132 (16733, 23532)	29% (24%, 34%)

Additionally, the gap between observed and counterfactual cases widened annually. Specifically, the relative reduction of EV71-infected HFMD was found to be 11% (95% CI: 6%, 16%) in 2017, while the reduction increased to 15% (95% CI: 10%, 20%) in 2018 and 66% (95% CI: 61%, 71%) in 2019, corresponding to 2776 (1531, 4021), 3503 (95% CI: 23,76, 4629) and 13,854 (95% CI: 12,796, 14,911) EV71-infected cases.

### 3.4 The effectiveness of EV71 vaccination program among different groups

**Fig. 3:**
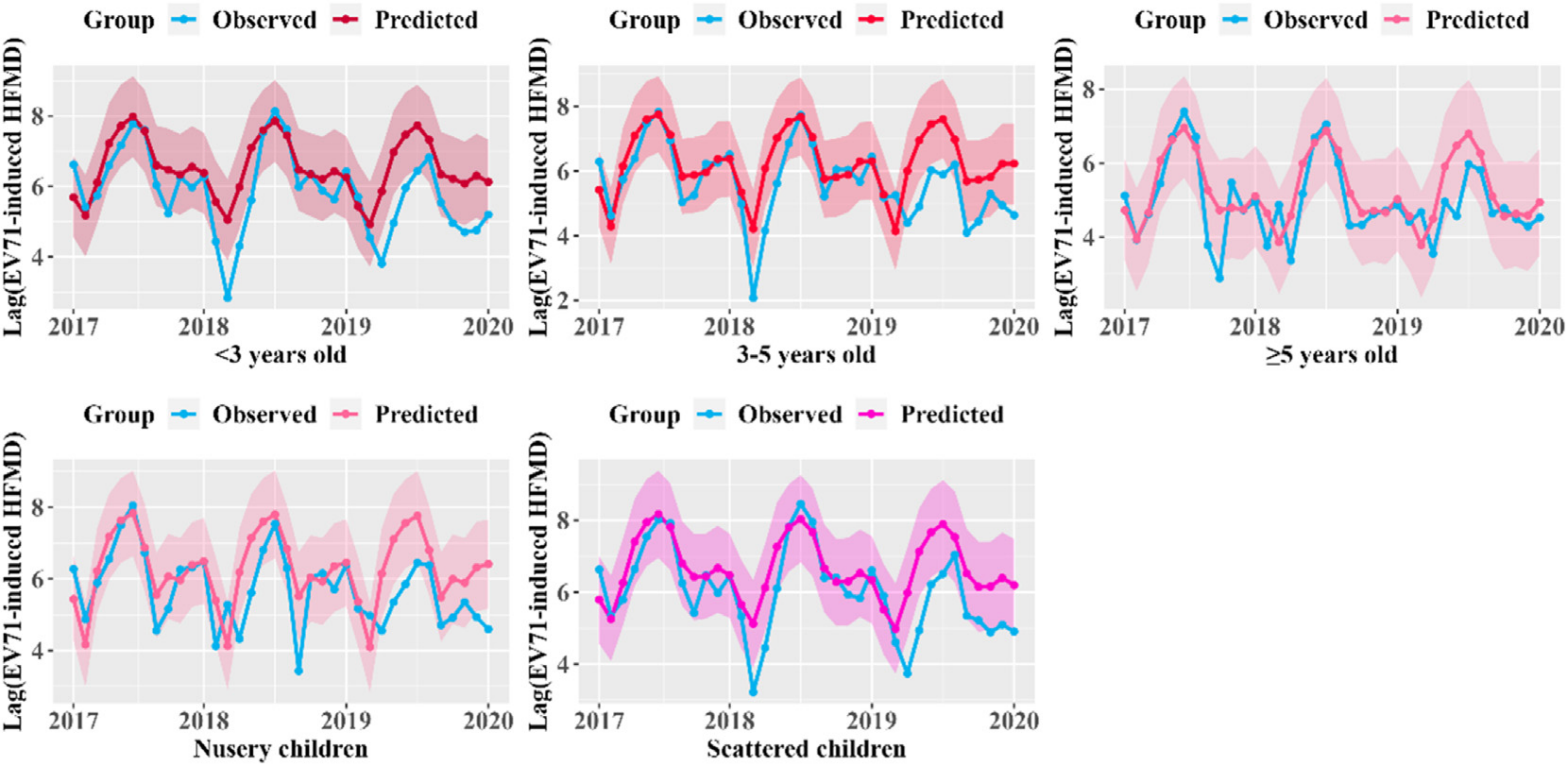
The comparison between monthly predicted counterfactual EV71-infected cases and observed cases (blue solid line) among different age and education level in Zhejiang Province, 2017–2019.

We also observed that the impact of the vaccination program was greater among those <5 years old compared with children ≥5 years old (Table 2 and Fig. 3). For example, the overall relative reduction during the study period was 32% (95% CI: 26%, 38%) and 35% (95% CI: 32%, 38%) in those <3 and 3–5 years old, which was twice as high as those aged >5 years (13%, 95% CI: 5%, 21%). Furthermore, the reduction of EV71 cases increased annually among those <3 and 3–5 years old. For the education groups, we estimated that approximately 38% (95% CI: 35%, 40%) and 26% (95% CI: 20%, 32%) EV71-infected HFMD cases could be prevent among nursery children and scattered children respectively (Table 2 and Fig. 3). Additionally, compared to the 2017–2018, all groups showed obvious increment in vaccine effectiveness in 2019.

**Table 2:** The relative reduction of EV71-infected cases among different age, education level and vaccination coverage rate in Zhejiang Province from 2017 to 2019.

Groups	2017	2018	2019	2017–2019
**Education level**				
Nursery children	5% (2%, 8%)	42% (39%, 44%)	69% (66%, 72%)	38% (35%, 40%)
Scattered children	19% (14%, 25%)	−2% (−8%, 3%)	67% (61%, 73%)	26% (20%, 32%)
**Age**				
<3 years old	23% (17%, 30%)	11% (5%, 17%)	67% (60%, 73%)	32% (26%, 38%)
3–5 years old	9% (6%, 13%)	27% (24%, 31%)	73% (69%, 76%)	35% (32%, 38%)
≥5 years old	−7% (−21%, 7%)	9% (1%, 17%))	49% (41%, 57%)	13% (5%, 21%)
**Vaccination coverage**				
Low coverage	−42% (−49%, −34%)	8% (0%, 15%)	63% (55%, 71%)	1% (−6%, 8%)
Moderate coverage	27% (22%, 32%)	−29% (−34%, −25%)	58% (53%, 62%)	19% (14%, 23%)
High coverage	26% (21%, 32%)	62% (56%, 68%)	76% (69%, 82%)	54% (48%, 61%)

**Fig. 4:**
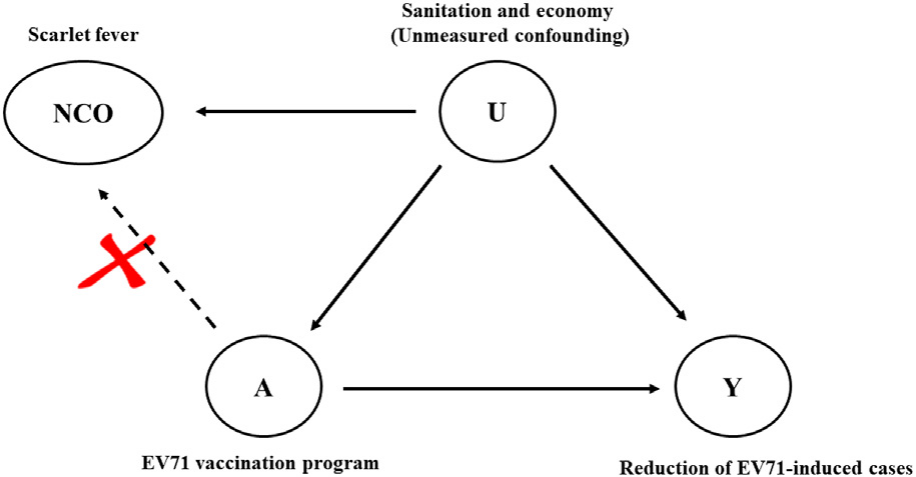
The negative control model: the causal effect of EV71 vaccination program (A) on reduction of EV71-infected cases (Y), subject to confounding by unmeasured sanitation and economy (U). The cases number of scarlet fever (W) is selected as NCO, which is not causally affected by A and was identified equal to A (equal U–A and U–Z additive association). Note: The red cross NCO should not directly associate with EV71-infected cases.

### 3.5 The effectiveness of EV71 vaccination in cities with various vaccination coverage rates

Moreover, we found that the cities with high vaccination coverage rate exhibited the highest reduction of EV71 cases and the reduction showed an upward trend over the years (Table 2 and Fig. 1C). Specifically, the relative reduction during study period was 1% (95% CI: 6%, 8%) in low vaccination coverage rate cities, 19% (95% CI: 14%, 23%) in moderate vaccination coverage rate cities and 54% (95% CI: 48%, 61%) for high vaccination coverage rate cities.

### 3.6 The results of negative control outcome model

**Fig. 5:**
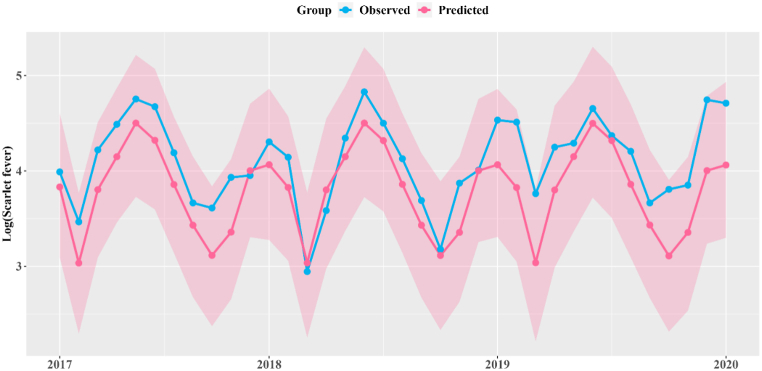
The comparison between monthly predicted counterfactual negative control cases (scarlet fever) and observed cases (blue solid line) from 2017 to 2019 in Zhejiang Province.

Using the NCO model, we investigated the association between EV71 vaccination and monthly scarlet fever (negative control) during 2017–2019 to detect the unmeasured confounding (Fig. 5 and Table S2). We calculated approximately −202 (95% CI: 504, 438), −150 (95% CI: 455, 513) and −303 (95% CI: 626, 394) cases were prevented in 2017, 2018 and 2019 respectively, indicating that the EV71 vaccination could not lead to a reduction in scarlet fever during 2017–2019. Therefore, the results of NCO model indicated that no unmeasured confounding factors were detected between vaccination and the reduction of EV71-infected HFMD.

### 3.7 Sensitivity analysis

According to the results of sensitivity analysis, our results remained stable under varying conditions. Firstly, the results of sensitivity analysis that included all severe cases as EV71 cases were consistent with our main findings (Table S3). Secondly, the results of sensitivity analysis that excluded patients older than 12 years, 10 years and 8 years was also similar to our original findings (Table S4).

## 4 Discussion

To our knowledge, this was the first study that combined the negative control outcome model with conventional methods to evaluate the real-world effectiveness of the EV71 vaccination, providing stronger statistical evidence for government to control the HFMD epidemic. Our findings demonstrated that the EV71 vaccination program resulted in a significant reduction in EV71-infected HFMD during the initial three-year period. Additionally, we detected no potential unobserved confounding in the association between vaccination and EV71 cases.

Aligned with previous findings, this study estimated that 29% reduction of EV71-infected HFMD could be attributed to the EV71 vaccination during 2017–2019. For instance, one study in Guangdong, China reported a reduction of approximately 41% in EV71-infected HFMD cases following the implementation of the vaccination program (Xiao et al., 2022). Similarly, a longitudinal study in Chengdu estimated that the vaccination program could prevent up to 60% of EV71 HFMD cases in 2017–2018 (Head et al., 2020). However, compared with the findings from Chengdu and Guangdong, the reduction rate of EV71-infected HFMD in our study was relatively lower. This discrepancy might be attributed to the different age structures and vaccination coverage rates. In detail, for Chengdu, the population in the study was 6–59 months with the coverage rate reached 54.3% in 2018, and the coverage rate of Guangdong have reached 35.8% in 2019. While, the coverage rate of Zhejiang Province were only 17% in 2019.

We also found that the gap between observed cases and predicted counterfactual cases increased by year, which could be explained by the increment of vaccination coverage rate in 2018–2019. In addition, we observed that cities with higher vaccine coverage rates provided a higher level of protection for their population, which was also consist with the previous findings (Takahashi et al., 2016). These findings emphasize the importance of increasing the EV71 vaccination coverage rate to effectively control the epidemic of EV71-infected HFDM.

Moreover, our study found that the vaccine effectiveness was greater among children aged 3–5 years compared to those under 3 years old, which was also consistent with previous studies (Cui et al., 2022; Wang, 2019). Notably, we observed a different pattern regarding the effect of the vaccine in different age groups. Specifically, in 2017, we observed a lower prevention effect for children aged 3–5 years, whereas in 2018, the prevention effect was higher. Conversely, for those under 3 years old, we found a lower prevention effect in 2018 and a higher prevention effect in 2017. The reason may be that during the period when the vaccine coverage rate had not yet reached a high level, the short-term outbreaks of HFMD could mask the effect of the vaccination program.

Similarly, we also observed a opposite pattern between nursery and scattered children across different educational groups. This distinction could be attributed to the Chinese school system, where children under the age of 3 are typically scattered, while those 3–5 years old are commonly enrolled in nursery institutions (Xiao et al., 2022). These results suggested that although low vaccination rate could achieve a certain degree of protection, it is insufficient to effectively control the increasing trend of HFMD cases in specific years. Thus, a further promotion of vaccination coverage was required.

Though no research to date has combined NCO with traditional methods to evaluate the effectiveness of EV71 vaccination, some studies have suggested that results solely from time-series data are difficult to detected unmeasured bias (Gasparrini et al., 2010; Skowronski et al., 2010). The findings of our study indicate that the EV71 vaccination program was not associated with negative control outcomes, indicating that there were no unmeasured bias (such as economic factors or healthcare policies) that directed associated with the negative control (the scarlet fever), suggesting that the reduction in EV71 cases can be causally attributed to the vaccination program.

There were two limitations that should be noted in our study. Firstly, the scarlet fever selected as the negative control outcome was not fully equivalent to EV71-infected HFMD, suggesting that our NCO model could only effectively detected the confounding bias, but cannot completely eliminate all unmeasured confounding factors (Fireman et al., 2009; Shi et al., 2020). Secondly, our study only focused on a relatively short-term period of three years (2017–2019) following the initiation of the vaccination program, and the results may be affected by short-term fluctuations in EV71 cases. Therefore, longer surveillance periods are needed to better estimate the effectiveness of the EV71 vaccination program.

## 5 Conclusion

This study observes that the number of EV71 cases in Zhejiang Province significantly decreased after the initiation of the vaccination program, with a greater reduction associated with higher vaccine coverage rate. Moreover, our study is also verifying a potential causal association between the vaccination program and the reduction of EV71 cases. These findings emphasize the importance of accelerating vaccine coverage and provide crucial statistical evidence to assist government officials in developing effective strategies to control the disease.

## Funding

This work was supported the grants from National Key R&D Program of China (2022YFC2305305), by grants from consultancy project (2022-JB-06) by the Chinese Academy of Engineering (CAE), and the Bill & Melinda Gates Foundation [Grant Number: INV-016826].

## Declaration of competing interest

The authors declare that they have no known competing financial interests or personal relationships that could have appeared to influence the work reported in this paper.
