# Supplementary material for: The impact of EV71 vaccination program on hand, foot and mouth disease in Zhejiang Province, China: A negative control study
Source: Infect Dis Model. 2023 Sep 5;8(4):1088–96. doi: 10.1016/j.idm.2023.09.001 (PMC10514095; doi:10.1016/j.idm.2023.09.001)
Supplement: Multimedia component 1 [file mmc1.docx]

**Table S1. The positive rates of EV71-infected HFMD among different groups**

| Date | <3 years old | 3-5 years old | >5 years old | Scattered children | Nursery children |
| --- | --- | --- | --- | --- | --- |
| 2017-1 | 0.256098 | 0.32967 | 0.233333 | 0.213483 | 0.213483 |
| 2017-2 | 0.193878 | 0.219512 | 0.206897 | 0.153226 | 0.153226 |
| 2017-3 | 0.282353 | 0.354839 | 0.277778 | 0.252747 | 0.252747 |
| 2017-4 | 0.408 | 0.43956 | 0.394737 | 0.356643 | 0.356643 |
| 2017-5 | 0.383886 | 0.590164 | 0.619718 | 0.445344 | 0.445344 |
| 2017-6 | 0.348387 | 0.46748 | 0.563025 | 0.342569 | 0.342569 |
| 2017-7 | 0.277439 | 0.366412 | 0.375 | 0.287411 | 0.287411 |
| 2017-8 | 0.162791 | 0.226415 | 0.069767 | 0.157658 | 0.157658 |
| 2017-9 | 0.069149 | 0.13913 | 0.030303 | 0.072464 | 0.072464 |
| 2017-10 | 0.095588 | 0.191257 | 0.215686 | 0.101307 | 0.101307 |
| 2017-11 | 0.096447 | 0.202703 | 0.114754 | 0.085714 | 0.085714 |
| 2017-12 | 0.124224 | 0.226415 | 0.136364 | 0.126316 | 0.126316 |
| 2018-1 | 0.041379 | 0.117647 | 0.076923 | 0.085366 | 0.085366 |
| 2018-2 | 0.027027 | 0.041667 | 0.085 | 0.033333 | 0.033333 |
| 2018-3 | 0.045455 | 0.073529 | 0.083333 | 0.046053 | 0.046053 |
| 2018-4 | 0.044674 | 0.067416 | 0.111111 | 0.061538 | 0.061538 |
| 2018-5 | 0.084404 | 0.064972 | 0.110465 | 0.091052 | 0.091052 |
| 2018-6 | 0.104895 | 0.133739 | 0.105 | 0.117816 | 0.117816 |
| 2018-7 | 0.074725 | 0.111111 | 0.058511 | 0.08274 | 0.08274 |
| 2018-8 | 0.031895 | 0.057692 | 0.027778 | 0.038168 | 0.038168 |
| 2018-9 | 0.042857 | 0.061224 | 0.025424 | 0.040073 | 0.040073 |
| 2018-10 | 0.048611 | 0.103659 | 0.057143 | 0.046584 | 0.046584 |
| 2018-11 | 0.052863 | 0.073333 | 0.066667 | 0.057692 | 0.057692 |
| 2018-12 | 0.098712 | 0.127551 | 0.054348 | 0.105882 | 0.105882 |
| 2019-1 | 0.086667 | 0.070707 | 0.057971 | 0.093168 | 0.093168 |
| 2019-2 | 0.075949 | 0.068 | 0.0569 | 0.069767 | 0.069767 |
| 2019-3 | 0.026549 | 0.066667 | 0.06 | 0.022901 | 0.022901 |
| 2019-4 | 0.034014 | 0.038674 | 0.083333 | 0.029412 | 0.029412 |
| 2019-5 | 0.047794 | 0.065041 | 0.02649 | 0.053628 | 0.053628 |
| 2019-6 | 0.053333 | 0.039216 | 0.063953 | 0.046632 | 0.046632 |
| 2019-7 | 0.068241 | 0.078431 | 0.072727 | 0.066253 | 0.066253 |
| 2019-8 | 0.042308 | 0.030769 | 0.061538 | 0.027439 | 0.027439 |
| 2019-9 | 0.028169 | 0.030675 | 0.087719 | 0.032922 | 0.032922 |
| 2019-10 | 0.02809 | 0.093023 | 0.083333 | 0.030457 | 0.030457 |
| 2019-11 | 0.046875 | 0.084337 | 0.097561 | 0.06015 | 0.06015 |
| 2019-12 | 0.092784 | 0.072289 | 0.133333 | 0.061947 | 0.061947 |

**Table S2. The observed, predicted, prevented cases of scarlet fever cases in Zhejiang Province, 2017-2019.**

| Year | Observed cases | Predicted cases | Prevented cases |
| --- | --- | --- | --- |
| 2017 | 785 | 583 (281, 1223) | -202 (-504, 438) |
| 2018 | 732 | 582 (277, 1245) | -150 (-455, 513) |
| 2019 | 885 | 582 (259, 1279) | -303 (-626, 394) |

**Table S3. The observed, predicted, prevented cases and the relative reduction of EV71-induced cases in Zhejiang Province, 2017-2019.**

| Year | Observed cases | Predicted cases | Prevented cases | Relative reduction |
| --- | --- | --- | --- | --- |
| 2017 | 23211 | 26111 (24882, 27340) | 2900 (1671, 4129) | 11% (6%, 16%) |
| 2018 | 20346 | 23381 (22279, 24484) | 3035 (1933, 4138) | 13% (8%, 18%) |
| 2019 | 7116 | 20927 (19877, 21976) | 13811 (12761, 14860) | 66% (61%, 71%) |
| 2017-2019 | 50673 | 70419 (67066, 73772) | 19746 (16393, 23099) | 28% (23%, 33%) |

**Table S4. The relative reduction of EV71-induced cases after exclude the participants over 12, 10 and 8 years old.**

| Groups | 2017 | 2018 | 2019 | 2017-2019 |
| --- | --- | --- | --- | --- |
| Age | 11% (6%, 16%) | 15% (10%, 20%) | 66% (61%, 71%) | 29% (24%, 34%) |
| Exclude over 12 years old | 11% (6%, 16%) | 14% (9%, 19%) | 67% (62%, 73%) | 29% (24%, 34%) |
| Exclude over 10 years old | 12% (7%, 17%) | 14% (9%, 19%) | 68% (63%, 73%) | 29% (25%, 34%) |
| Exclude over 8 years old | 15% (10%, 20%) | 13% (8%, 17%) | 69% (64%, 74%) | 30% (25%, 35%) |


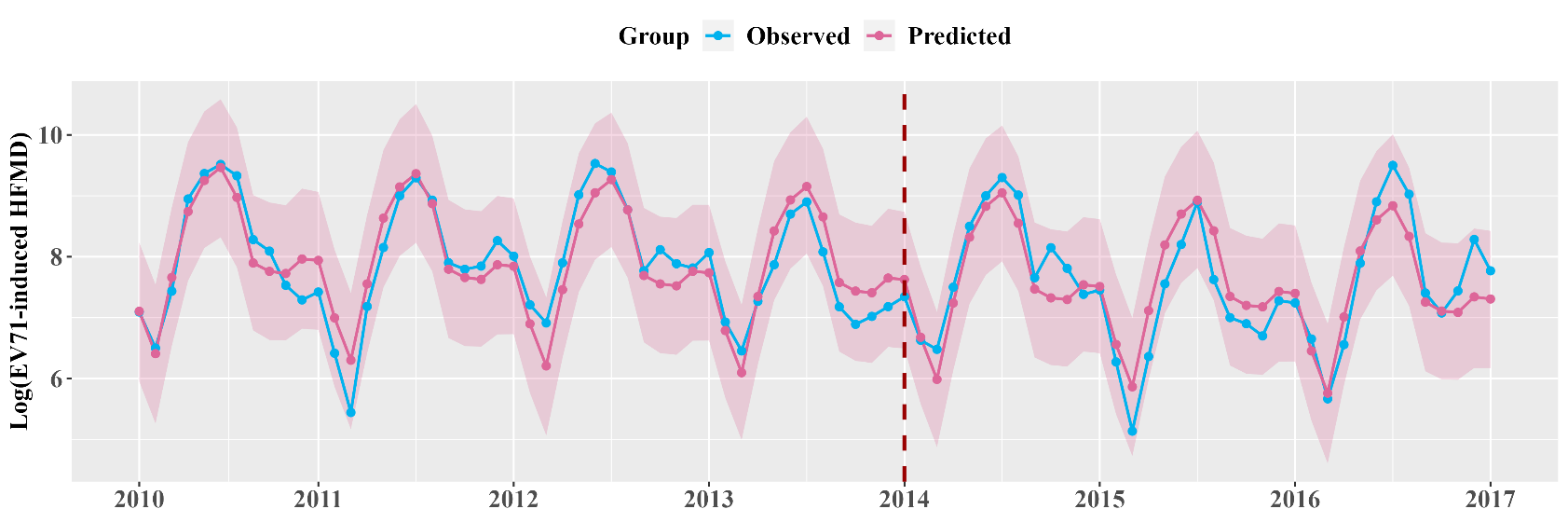


**Fig S1. The observed and predicted cases of EV71-induced cases in Zhejiang Province from 2010-2017, by using the dataset of 2012–2014 as training data.**


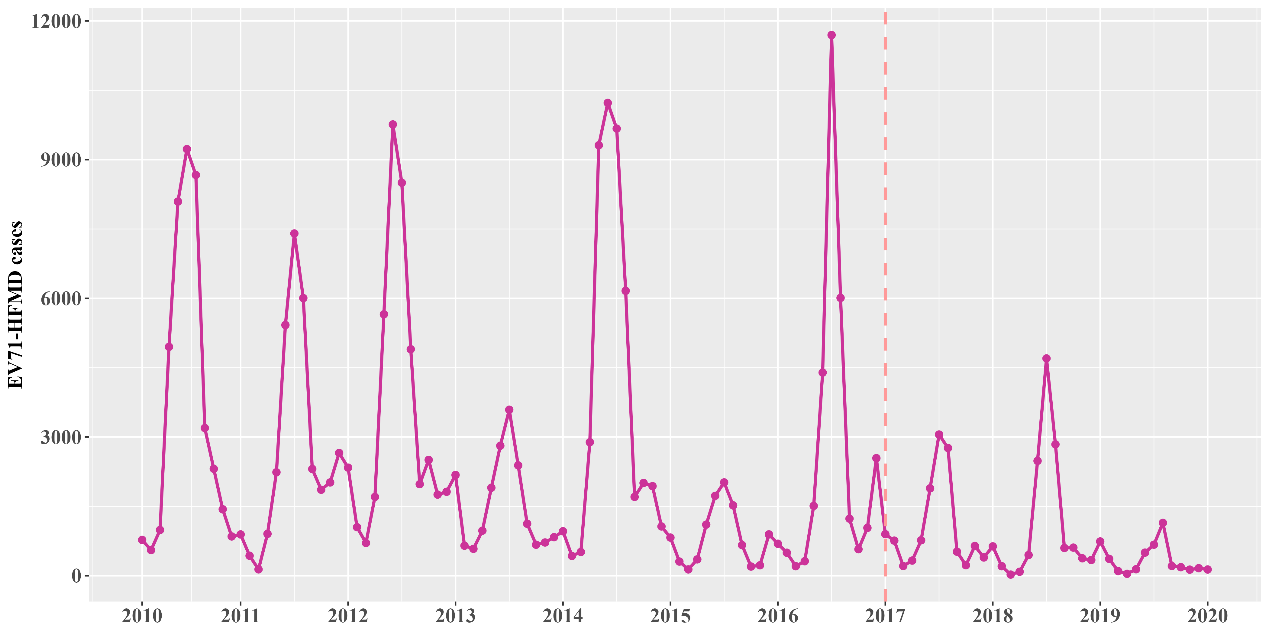


**Figure S2. The temporal distribution of EV71-related cases for scattered children.**


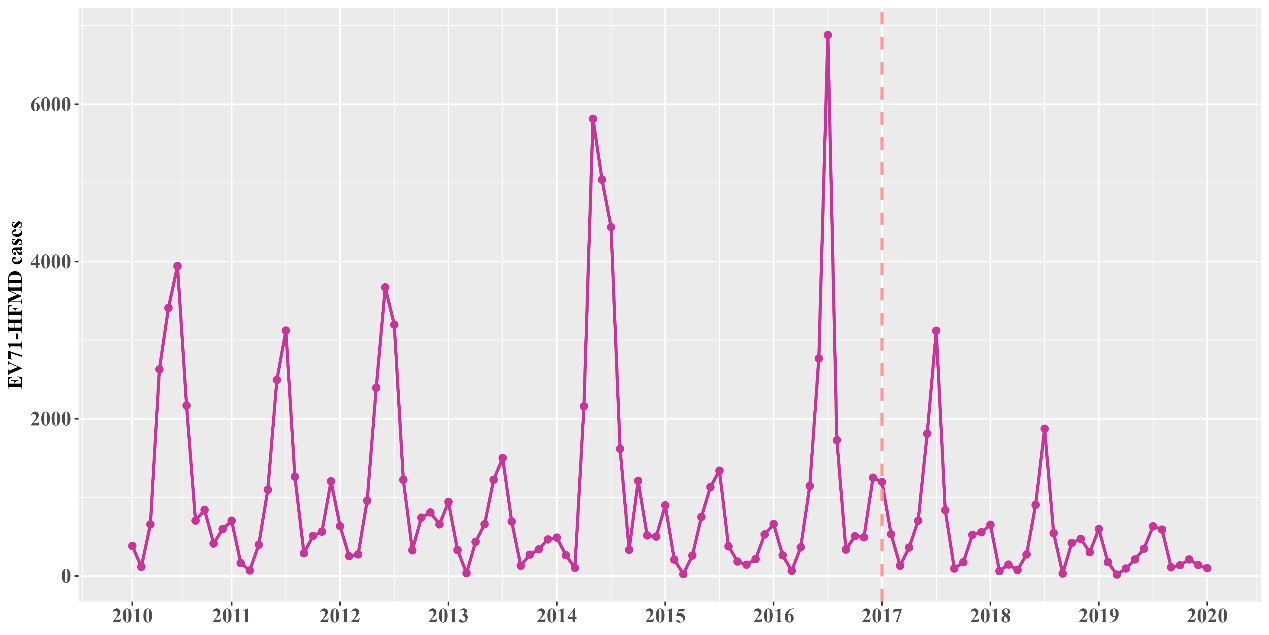


**Figure S3. The temporal distribution of EV71-related cases for nursery children.**
